# Viruses of Microbes 2020: The Latest Conquest on Viruses of Microbes

**DOI:** 10.3390/v13050802

**Published:** 2021-04-30

**Authors:** Tessa E. F. Quax, Marianne De Paepe, Karin Holmfeldt

**Affiliations:** 1 Archaeal Virus-Host Interactions, Faculty of Biology, University of Freiburg, Schaenzlestrasse 1, 79104 Freiburg, Germany; 2 Université Paris-Saclay, INRAE, AgroParisTech, Micalis Institute, 78350 Jouy-en-Josas, France; marianne.depaepe@inra.fr; 3 Centre for Ecology and Evolution in Microbial Model Systems (EEMiS), Department of Biology and Environmental Science, Faculty of Health and Life Sciences, Linnaeus University, SE-39231 Kalmar, Sweden; Karin.holmfeldt@lnu.se

This Special Issue celebrates viruses of microbes: those viruses that infect archaea, bacteria and microbial eukaryotes. This issue is associated with the 6th Viruses of Microbes (VoM) conference in Guimarães (www.vom2020.org, accessed on 1 April 2021), Portugal, that was originally planned for the summer of 2020, but has, in light of the pandemic, been postponed to 18–22 July 2022. 

The International Society for Viruses of Microbes (ISVM) supports the biannual organization of the VoM meetings and unites researchers worldwide that study the different aspects of viruses of microbes, ranging from structural biology to ecology and from applied to fundamental science (www.isvm.org, accessed on 1 April 2021). This active and expanding research community benefits not only from the scientific exchange during the VoM meetings, but also online, such as recently during the successful virtual seminar series, iVoM, that was organized by the local organizers of the 6th VoM. The meetings and online seminar series highlight the most recent developments in the field and thus have on display “The latest conquests on Viruses of Microbes”. 

Studies of viruses of microbes have driven many major scientific revolutions, such as the usage of phage (derived) therapy to fight antibiotic resistance, the development of molecular biology, the discovery of the major impact of viruses on biochemical cycling and the recently, Nobel-awarded, adaptation of CRISPR-Cas for genome editing. Comparison between the virion structures, genomes and infection strategies of different viruses of microbes enables a unifying view of viruses and helps to understand how viruses originated, how they develop and how they impact life on earth. 

In this Special Issue, recent advances in the field of microbial virology are collected. Two reviews summarize the novel developments in understanding RNA viruses of aquatic eukaryotes [1] and on entry mechanisms of prokaryotic viruses [2]. Comparison between different viruses requires genomic evaluation tools, such as the VIRIDIC tool presented in this issue [3]. Such tools, in combination with omics approaches [4] can support study of the role of individual viral proteins in the viral infection cycle [5] and manipulation of the host cell [6,7,8]. Deciphering the role of viral proteins is important for our understanding of viral infection strategies, including host recognition [9]. Isolation of novel viruses from underexplored environments is still a prerequisite for further exploration of the viral gene repertoire and molecular characterization of viral infection [10,11].

The combination of efforts, such as those bundled in this Special Issue, will allow us to entangle the strategies that individual viruses use to propagate and the mechanisms by which they impact life from individual cells to ecosystems. Microbial viruses were discovered over hundred years ago, and the more we learn about them, the more they keep surprising us. It is now an exciting time for microbial virologists, and this growing research community will surely contribute to unlocking many more secrets of these fascinating viruses. 

## Data Availability

Not applicable.
